# Editorial

**Published:** 1900-11-15

**Authors:** 


### EDITORIAL.

### DENTISTRY A SPECIALTY OF MEDICINE.

In the October *International Dental Journal* the editor replies to our editorial comment, in the July issue, on his attitude toward the suggestion that Dentistry shall be practiced as a specialty of Medicine under the Medical Doctors degree, as set forth by the essayists in the section on Stomatology of the American Medical Association. The editor of the *International Journal* intimates that we are influenced in our position by personal preference which has not only caused us to misinterpret his position, but to take a somewhat restricted view of the question.

Now we acknowledge that we have a very strong preference for the Doctor of Dental Surgery degree; because it symbolizes so much that is woven into our life. For more than a quarter of a century we have labored to the best of our ability to interpret its meaning by devotion to its doctrines and duties, and this devotion has engendered a respect for it second to no other. And our loyalty to it as one of the noble callings of life seems to increase with the passing of time. Perhaps it is this sentimental respect, or loyalty, which caused us to resent the intimation that our degree was not as respectable as some others; or that it was too narrow for the requirements of its present exacting and enlarged spheres of usefulness. If so, we do not feel called upon for a defense as this is a somewhat personal factor, but still pertinent.

Again we acknowledge that a spirit of resentment—doubtless begotten of narrow prejudice, yet none the less genuine—moved us to protest; when our good friend and honored co-laborer who for so long has been identified with the dental profession, giving unsparingly of his physical and mental resources to its development as a separate and distinct department of the healing art, as a faithful practitioner, a teacher of many years and a leader of thought in its literature and places of associate effort, now lending his voice and influence to develop a sentiment fostered by a small minority of our profession who have seen fit to qualify themselves as Medical Doctors for practice, and who seek to submerge the Dental Degree and its significant present and glorious past in a great "Medical Trust." And to whom shall be the benefit?

It may be that the knowledge acquired in obtaining the medical degree and practicing under it has so enlarged their horizon, that our medically educated practitioners can see farther into the future than we, or it has put them in possession of some convincing arguments that will cause us to see this matter in a more favorable light; if so, we will try to open our minds and with our limitation be convinced; but we want something that comes from actual experience or that will stand the test of a reasonable logical scrutiny. For we are, in spite of our prejudices, earnestly seeking the advancement and enlargement of the sphere of usefulness of our profession. We realize that to medical science we owe much for the present status of the profession. From clinical medicine we have also drawn much, and shall in the future need to keep constantly in touch with its advances, but we are not yet ready to be absorbed by it or to accept it as the *sine qua non* as a qualification for the practice of dentistry. In our former editorial we hinted only at some of the facts which we can not see any way of overcoming, but which it will be absolutely necessary to change before this consolidation can be accomplished.

We have purposely confined our remarks at this time to the sentimental reasons, which, if we are not mistaken, will prove the most difficult factor to be dealt with. Whether this sentiment is narrow and bigoted or not, it is real, and is a potent influence in holding dental practitioners together in a dignified and respected profession. And we seriously doubt the advisability of abandoning this independence of thought and action lest it kill the spirit. For argue as we may personal preference, intelligent or otherwise, is a cherished privilege as well as a mighty force and ought to be taken into account in deciding a question of so much importance.

### JUST SEE WHAT WE ARE COMING TO.

At the last meeting of the American Medical Association the following resolution was offered in the Section on Stomatology as an encouragement to higher education and original research:

That the Section of Stomatology of the American Medical Association take steps to acquire the power or authority, whether by charter or otherwise, by virtue of which it may be empowered to confer upon deserving individuals, regardless of sex, the degree of S.D.,—*Stomatologiæ Doctor* (Doctor of Stomatology); and in order that this degree may stimulate to higher education and original research, would recommend that this degree be conferred strictly and only upon compliance with the following requirements:

1st. That the recipient shall be of good moral character and in good professional standing.

2nd. That he shall have previously acquired the degree of M.D. from an institution not conferring the degree of M.D., subsequent to 1903, upon less than the requirement of an attendance upon four sessions, of not less than seven months each, in four different years.

3rd. That he shall also have taken a course upon the mouth and its associate parts.

4th. That he shall have made some original and meritorious contribution of especial value to stomatology.

5th. That this degree shall not be conferred except upon the unanimous vote in the affirmative of *all* the members present at the meeting at which this action is contemplated.

The following form of diploma was suggested by Dr. Walker as a suitable certificate:

AMERICAN MEDICAL ASSOCIATION—SECTION ON STOMATOLOGY.

*To all those to whom these presents shall come, greeting.*

Know ye that, by virtue of the authority vested in the Section on Stomatology of the American Medical Association, we, the members voting unanimously in the affirmative, do this day confer upon X . . Y . . Z . . M.D., the degree of S.D.—*Stomatologiæ Doctor*—in recognition of his original, meritorious, and valuable contributions to the sum of the scientific knowledge concerning the mouth, he having complied with the following requirements, to-wit: That no person shall receive this degree except that, first, he shall have previously acquired the degree of M.D.; second, that he shall also have taken a course upon the mouth and its associate parts; third, that he shall have made some original, meritorious and valuable contribution to stomatology, and fourth, that it shall have been unanimously voted by the members present that the degree be conferred upon him.

Wherefore, we, the members of the section, have caused to be affixed hereunto the signatures of the acting chairman of the section, and of the three last retired chairmen, and also of the present secretary of the section; the same to be stamped with the seal of the Section on Stomatology of the American Medical Association.

### NOTICE.—NEW COLLEGES.

*To Whom it May Interest:*—At the annual meeting of the National Association of Dental Faculties, held at Niagara Falls, 1899, the following action was had:

*Resolved,* "That a commission, consisting of three persons, be appointed, whose duty it shall be to take cognizance of, investigate and advise with any parties contemplating the establishment of a new college or the re-organization of an old one."

In the performance of the duties of this commission it shall be competent to take into consideration the following points, viz:

All the circumstances that attach to it; the motive that prompts such an organization; the need for it; the proposed locality; the character and ability of those who propose to conduct it; the resources that may be available for its establishment, and any other points that may have a bearing, for or against the establishment of a proposed college.

The attainment of full knowledge on these points would enable the commission to advise wisely.

Any party or parties having in contemplation the organization of a new dental college, or the re-organization of one already in existence are requested to communicate with this commission for conference.

It shall be the duty of this commission to report to this body at each annual meeting; giving in detail such facts and conditions pertaining to the subject as the commission may find.

J. Taft, *Chairman*, Cincinnati, O.
Geo. E. Hunt, Indianapolis, Ind. } *Commission.*
Frank Holland, Atlanta, Ga.

We are very glad to give space to this notice. It is to be hoped that the object sought to be covered by this commission, may be attained. Too many colleges have been launched by persons who little realized what sacrifice of time and money would be needed to put such institutions on a successful educational basis. Consequently they have drifted into commercial rather than educational institutions.

### THE OHIO STATE DENTAL SOCIETY.

Before another issue the Ohio State Dental Society will have met and the results of another year of progress will be recorded. Preparations are actively being made for a great meeting. The program is not yet ready for announcement, but it will be a good one and will include some special features by men from other States. The program assures a good attendance, and the larger the attendance the more profitable, especially should each one contribute something. As announced, the meeting will be held December 4th, 5th and 6th at the Great Southern Hotel in Columbus, Ohio.

